# Factors affecting the radiation response in glioblastoma

**DOI:** 10.1093/noajnl/vdac156

**Published:** 2022-09-25

**Authors:** Radhika Aiyappa-Maudsley, Anthony J Chalmers, Jason L Parsons

**Affiliations:** Department of Molecular and Clinical Cancer Medicine, University of Liverpool, William Henry Duncan Building, Liverpool, L7 8TX, UK; Institute of Cancer Sciences, College of Medical Veterinary and Life Sciences, University of Glasgow, Glasgow, UK; Department of Molecular and Clinical Cancer Medicine, University of Liverpool, William Henry Duncan Building, Liverpool, L7 8TX, UK; Clatterbridge Cancer Centre NHS Foundation Trust, Clatterbridge Road, Bebington, CH63 4JY, UK

**Keywords:** Carbon ions, DNA damage repair, glioblastoma, ionizing radiation, proton beam therapy

## Abstract

Glioblastoma (GBM) is a highly invasive primary brain tumor in adults with a 5-year survival rate of less than 10%. Conventional radiotherapy with photons, along with concurrent and adjuvant temozolomide, is the mainstay for treatment of GBM although no significant improvement in survival rates has been observed over the last 20 years. Inherent factors such as tumor hypoxia, radioresistant GBM stem cells, and upregulated DNA damage response mechanisms are well established as contributing to treatment resistance and tumor recurrence. While it is understandable that efforts have focused on targeting these factors to overcome this phenotype, there have also been striking advances in precision radiotherapy techniques, including proton beam therapy and carbon ion radiotherapy (CIRT). These enable higher doses of radiation to be delivered precisely to the tumor, while minimizing doses to surrounding normal tissues and organs at risk. These alternative radiotherapy techniques also benefit from increased biological effectiveness, particularly in the case of CIRT. Although not researched extensively to date, combining these new radiation modalities with radio-enhancing agents may be particularly effective in improving outcomes for patients with GBM.

Glioblastoma (GBM and WHO grade 4) is the most common primary brain tumor in adults with an annual worldwide incidence of ~170 000 cases.^1^ The 2021 (5th edition) classification of GBM by the World Health Organization (WHO) classifies all IDH-wild-type tumors as primary GBMs, and all IDH mutant tumors as astrocytomas.^2^ The current treatment for newly diagnosed GBM is neurosurgery followed by radiotherapy (60 Gy delivered in 2 Gy fractions over 6 weeks) with concomitant temozolomide (TMZ) treatment, which is an alkylating agent that penetrates the blood–brain barrier (BBB).^3^ This regimen is then followed by TMZ maintenance therapy for 6 months.^4^ However, GBMs are inherently resistant to conventional therapies, and even with the clinically approved treatment, the vast majority of patients experience tumor recurrence or progression that is ultimately responsible for their death. Median survival is, therefore, poor at 12–15 months, and only 5%–7% of patients survive beyond 5 years.^5^ No effective treatment strategy has yet been established for recurrent or progressive disease, but current options include surgery, re-irradiation, systemic therapies, and palliative care.^6^

Prognostic factors for patients with GBM include the Karnofsky Performance Score and the extent of resection of the tumor tissue. Complete surgical excision is not possible because of the invasive and diffuse nature of GBMs. However, surgical removal of ~70%–98% of tumor tissue, when possible, is associated with improved prognosis, although bulky residual tumors can negatively influence prognosis. An important molecular biomarker in GBM is the methylation status of the O^6^-methylguanine-DNA methyltransferase (MGMT) promoter, which can predict response to treatment as well as overall prognosis. Epigenetic silencing of the MGMT promoter suppresses transcriptional expression of the protein, which normally functions to remove O^6^-alkyl groups from guanine bases in DNA thus counteracting the effects of alkylating agents, such as TMZ. Therefore, patients whose tumors exhibit methylated MGMT promoters have a better prognosis and respond relatively well to TMZ in combination with radiotherapy, compared to unmethylated patients who derive no benefit from this treatment modality.^7^

The aim of this review is to provide an overview of the key biological factors and processes known to influence the efficacy of radiotherapy as a treatment for GBM. We then cover some of the strategies being explored to enhance the sensitivity of GBM to radiotherapy, particularly through targeting the cellular DNA damage response (DDR). Finally, we discuss the potential for other types of radiotherapy, including proton beam and carbon ion therapy (PBT and CIRT) to optimize treatment and ultimately improve outcomes.

## Factors Affecting GBM Treatment

### DNA Damage Response

**Figure 1.**
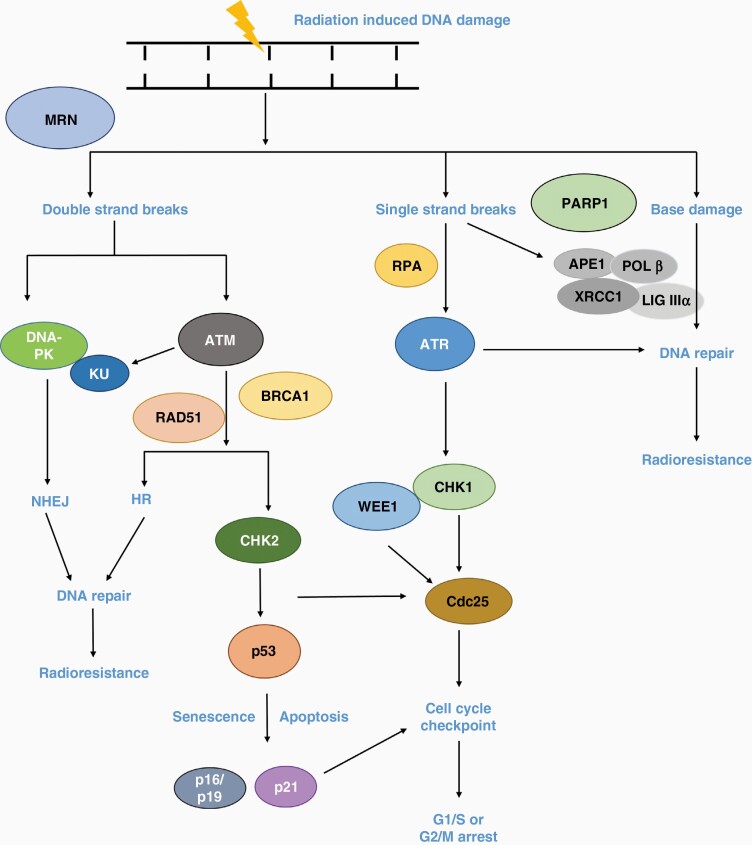
IR-induced DNA damage response (DDR) pathways. DNA-PK and ATM preferentially activate the NHEJ and HR pathways, respectively. Both CHK1 and CHK2 phosphorylate cell division cycle 25 (CDC25) which influences the G1/S and G2/M checkpoints. WEE1 phosphorylates CDC25 which prevents progression into mitosis. Activation of p53 by the ATM-CHK2 cascade promotes p21 transcription, which arrests cells at G1/S phase or can stimulate apoptosis or cellular senescence. In terms of direct SSBs and those generated through intermediates of BER, PARP1 binds and promotes further repair through DNA polymerase β (POLβ) and X-ray repair cross-complementing 1 (XRCC1) in complex with DNA ligase IIIα. ATR is activated by stalled replication forks and through replication protein A (RPA) bound to single-stranded DNA.

The DDR is involved in the maintenance of genome integrity and stability by correcting damaged DNA (Figure 1). In mammalian cells, the major repair pathways that are activated in response to DNA damage are base excision repair (BER), which resolves DNA base damage and single-strand breaks (SSBs), plus nonhomologous end joining (NHEJ) and homologous recombination (HR) that repair DNA double-strand breaks (DSBs). DSBs are the most lethal lesions induced by ionizing radiation (IR), although complex DNA damage (CDD) containing multiple DNA lesions within close proximity also contributes to radiation-induced cell death.^8^ DDR signaling responses are controlled by the protein kinases ataxia telangiectasia-mutated (ATM) and ataxia telangiectasia and Rad3-related (ATR), which activate cell cycle arrest through the checkpoint kinases CHK2 and CHK1, respectively. Other key enzymes involved in DNA repair include the DNA-dependent protein kinase (DNA-PK), which coordinates NHEJ, and the DNA strand break binding protein poly (ADP-ribose) polymerase 1 (PARP1) involved predominantly in SSB repair but also in a sub-pathway of NHEJ.

Research has reported that GBM cells demonstrate enhanced G2/M checkpoint activation and DNA repair in response to IR, both of which counteract the cytotoxic effects of the treatment.^9^ Several DDR proteins, including ATM, ATR, CHK1, PARP1, and RAD51 are upregulated in GBM stem-like cells (GSCs) and enzyme activation appears to be primed even in unirradiated cells, leading to enhanced repair and radioresistance.^10^ Inhibiting RAD51, a key factor in the HR pathway, has been demonstrated to increase apoptosis, decrease cell survival, and delay the repair of IR-induced DNA damage in GSCs.^11^ In GBM, mutations in the mismatch repair (MMR) genes are implicated in TMZ resistance. However, a CRISPR-Cas9 screen in patient-derived GBM cells identified that the core members of the mismatch repair (MMR) pathway, MLH1, MSH2, MSH6, and PMS2 were enriched in TMZ resistant cells, after 3 weeks of treatment with TMZ.^12^ Such findings emphasize that alterations in the DDR play a key role in the resistance of GBM to current treatments.

### Glioblastoma Stem Cells

The presence of Glioblastoma Stem Cells (GSCs) that have the capacity to self-renew and differentiate into multi-lineages has been implicated in the propensity of GBM to recur after treatment, and hence in the poor patient outcomes observed. DNA repair pathways are predominantly activated in GSCs and increased levels of CD133^ + ^stem cells are evident in recurrent tumor samples after treatment with high-dose radiotherapy.^13^ Stem cell markers including CXCL12, CD133, SOX2, OLIG2, NANOG, aldehyde dehydrogenase 1 (ALDH), NESTIN, and Integrin α6 have been shown to be expressed in GSCs.^14^ Expression of the transcription factor forkhead box M1 (FoxM1) is associated with radioresistance and maintenance of GSCs via increased expression of SOX2, the master regulator for maintaining stem cells in an undifferentiated state. Moreover, overexpression of SOX2 has been associated with increased clonogenic survival and induces a GSC-like phenotype in tumor cells. Inhibiting FoxM1 in combination with IR was shown to reduce SOX2 expression and impair tumor growth in GBM xenograft models, suggesting that the FoxM1-SOX2 pathway is involved in the radiation response of GBM.^15^ Knockdown of the NOTCH1 transmembrane signaling receptor has also been reported to increase the radiosensitivity of GSCs,^16^ while decreasing the adaptive response of GBM xenografts in hypoxia.^17^

### Hypoxia and the Tumor Microenvironment

In GBM, the partial pressure of oxygen has been reported to be around 13mmHG.^18^ Hypoxia, defined as a reduction in tissue oxygen levels (<1%–2% O_2_), is associated with tumor aggressiveness, poor patient survival, and radiotherapy resistance in GBM.^19^ As well as causing radioresistance directly, the presence of hypoxia in GBM promotes invasion and metastasis of tumor cells into the surrounding brain tissue to evade the hypoxic tumor microenvironment.^20^*In vitro* studies using GBM cell lines have demonstrated that reducing the oxygen concentration to 9% was sufficient to stimulate tumor cell migration, induce hypoxia-related genes and cause therapy resistance.^21^ Tumor cells adapt to hypoxia by upregulating neovascularisation, or growth of new blood vessels, to ensure an adequate supply of oxygen and nutrients to the rapidly proliferating tumor. However, this creates a more hypoxic environment because the new blood vessels formed are leaky, small, and blocked, which further leads to blood flow disturbances and prevents immune cell infiltration. The presence of tumor hypoxia also reduces the efficacy of chemotherapeutic agents by preventing drugs from reaching and targeting hypoxic areas of tumors. The acidic pH in the hypoxic tumor microenvironment can also inactivate certain pH-sensitive agents. All these favor the emergence of therapy-resistant, aggressive and metastatic clones.^22^ Furthermore, formation of new blood vessels leads to tumor permeation and formation of micrometastasis.^23^ Levels of the transcription factor hypoxia-inducible factor 1α (HIF-1α) are also related to radiation effects on the tumor microenvironment. HIF-1α can initiate an adaptive response to IR through upregulation of vascular endothelial growth factor (VEGF), which promotes survival of endothelial cells.^24^ This has led to several studies assessing the efficacy of combining antiangiogenic agents with IR to promote the radiosensitivity of endothelial cells. Bevacizumab (Avastin), a monoclonal VEGF antibody was evaluated in 2 large phases III trials (LB-05 and EORTC 26101) but reported no significant benefit of adding this agent to standard GBM treatment. Despite seeing no survival advantage, bevacizumab was approved for the treatment of recurrent GBM in the United States and other countries.^25^ The NCT05284643 trial has recently been initiated to investigate the efficacy of spectroscopic magnetic resonance imaging, in combination with proton and Avastin in GBM patients.

### Autophagy

Autophagy refers to the lysosomal-mediated degradation of unwanted and nonessential cellular components, and has been recognized as a survival mechanism that cells use to adapt to hostile environments including hypoxia and in response to IR. GBM cells have been reported to employ autophagy to reutilize unwanted or damaged proteins to aid in the progressive growth of the tumor.^26^ Exposure of CD133^ + ^GSCs to IR was found to activate autophagy, whereas inhibition of autophagy using the autophagic inhibitor bafilomycin A1, caused radiosensitization and decreased the ability of GSCs to form neurospheres.^27^ Cathepsin D is a class of cysteine proteases that demonstrates a positive correlation with autophagic markers such as LC3A and LC3B in GBM. Overexpression of cathepsin D has been associated with radioresistance and poor survival of GBM patients, and inhibiting cathepsin D by small interfering RNA (siRNA) or by the inhibitor pepstatin-A was found to suppress autophagy and sensitize GBM cells to IR.^28^ These data point to the involvement of autophagy in the radioresistant phenotype of GBM.

### Altered metabolism

GBM cells display alterations in various metabolic pathways that help them adapt to and proliferate in the tumor microenvironment, and to survive after treatment. For example, purine metabolites such as guanylates have been associated with increased DNA repair and therapy resistance^29^ and purine overexpression has also been associated with poor patient prognosis.^30^ Inhibiting purine synthesis using mycophenolic acid (MPA) was found to sensitize GBM cells and neurospheres to IR through delays in DSB repair, whereas nucleoside treatment before IR increased radioresistance. However, interestingly, this study found that depleting pyrimidines using teriflunomide did not radiosensitize or affect the ability of GBM cells to repair IR-induced DSBs, suggesting that only purine metabolites are responsive to IR. High expression of inosine monophosphate dehydrogenase 1 (IMPDH1), which is involved in purine synthesis, has been associated with shorter survival in GBM patients.^29^ Clinical assessment of mycophenolate mofetil (MMF), the oral prodrug of MPA, in combination with IR is currently being investigated in GBM patients (NCT04477200). In addition to purines, GBM has been observed to have a higher concentration of lipid droplets compared to normal tissues. Inhibiting monoacylglycerol lipase, responsible for converting monoglycerides into fatty acids, was found to inhibit GBM cell proliferation.^31^

## Combinatorial Strategies for GBM

As described above, the highly radioresistant nature of GBM has been attributed to in part the presence of GSCs that promote both activations of the G2/M checkpoint and efficient DNA repair. Therefore, novel treatments to enhance IR have focused on targets within DDR pathways, but also through particle ions that are less affected by factors such as hypoxia and the tumor microenvironment.

### ATM Inhibitors

ATM is a member of the phosphoinositide 3-kinase (PI3K)-related protein kinase family (PIKKs) and upon sensing DNA damage, ATM is rapidly recruited to sites of DSBs through interaction with the MRE11-RAD50-NBS1 (MRN) complex.^32^ The radioresistant nature of GSCs has also been attributed to increased basal levels and activation of ATM, and whilst the first generation of ATM inhibitors such as KU60019 were observed to be effective radiosensitizers, they were not capable of crossing the BBB.^33^ However, the novel oral ATM inhibitor, AZD1390 has demonstrated a 6-7-fold higher brain penetrance than the previous generation of inhibitors such as AZD0156. It has been shown that nanomolar concentrations of AZD1390 were sufficient to inhibit ATM in p53 mutant LN18 GBM cells, and in combination with IR this increased G2 phase cell cycle arrest, apoptosis, and micronucleus formation. Radiation dose enhancement values associated with AZD1390 in p53 mutant LN18 cells and patient-derived xenograft (PDX) models were approximately 3-fold higher than in p53 wild-type models. In an orthotopic lung-brain metastatic model, the combination of AZD1390 and IR significantly decreased tumor volume and increased animal survival compared to IR treatment alone.^34^ This exciting new agent is currently being evaluated in combination with IR in the treatment of brain cancers, primarily GBM (NCT03423628).

### ATR Inhibitors

ATR is of particular interest because it causes apparent desensitization of GBM cells to treatment with TMZ. TMZ is known to activate the ATM-ATR signaling pathway in cells expressing low levels of MGMT protein, and therefore treating MGMT-deficient GBM cells with TMZ has been shown to increase sensitivity to ATR inhibitors. Additionally, siRNA knockdown of ATR increased TMZ-induced GBM cell death and inhibition of CHK1 led to the same response, indicating that the ATR-CHK1 pathway is an attractive therapeutic target in GBM.^35^ In GSC models, the ATR-CHK1 pathway is also activated by IR, and inhibition of either ATR or CHK1 was found to increase mitotic catastrophe resulting in radiosensitization.^10^ Building on these observations, a recent study investigated the efficacy of the ATR kinase inhibitor, berzosertib in GBM cells and was demonstrated to enhance the sensitivity of U87 and U251 cells and a PDX cell line, GBM22 (p53 mutant, MGMT methylated), to TMZ. However, the compound was actively effluxed at the BBB, leading to low free drug concentrations in the brain tissue and in intracranial PDX models. In keeping with this, berzosertib failed to improve survival in PDX-bearing mice when given in combination with TMZ.^36^ Alternative ATR inhibitors with superior brain penetrance are therefore needed for clinical trials to advance.

### DNA-PK inhibitors

DNA-PK is an important component of the NHEJ repair pathway, which is the dominant DSB repair pathway, particularly in the G1 phase when HR is nonfunctional. Overexpression of DNA-PK in 57.2% gliomas was associated with shorter survival of patients, and the inhibitor KU0060648 has been reported to significantly sensitize U87 and M059K GBM cells to TMZ.^37^ Another study discovered and characterized a novel DNA-PK inhibitor, nedisertib (M3814), and reported its potent radiosensitizing activity in a dose-dependent manner in the DNA-PK proficient MO59K GBM cell line where the enhancement ratio, calculated at 10% survival, ranged between 2.5 and 6. However, no enhanced radiosensitization was observed in the DNA-PK deficient M059J GBM cell line demonstrating the specificity of the drug to its target.^38^ Although effective in preclinical evaluation, there is a substantial risk that inhibition of DNA-PK catalytic activity will severely exacerbate normal brain toxicity. This is based on the knowledge that leukemia patients with deficiencies in DNA ligase IV, a core component of the NHEJ pathway, encountered fatalities when treated with radiotherapy.^39^ However, it is unclear whether transient inhibition of DNA-PK would have the same effect, and if this can be combined with radiotherapy to achieve greater tumor control. Nedisertib is currently being trialed clinically in combination with IR and TMZ in GBM patients with newly diagnosed unmethylated MGMT status (NCT04555577). Additionally, the INSIGhT trial is investigating CC-115, the dual inhibitor of DNA-PK and mammalian target of rapamycin, in combination with IR in newly diagnosed patients with unmethylated MGMT (NCT02977780) to assess if targeting these dual pathways will have greater anticancer efficacy.

### PARP Inhibitors

PARP inhibitors block the enzymatic activity of PARP, and some (eg, talozoparib) increase formation of trapped PARP-DNA complexes resulting in unrepaired DNA damage and cell death. PARP inhibitors are known to act as modest radiosensitizers across a broad range of cancer models, including GBM, and are highly potent sensitizers to TMZ, hence they have exciting potential in this disease. The currently available PARP inhibitors have different pharmacokinetic properties with regard to distribution across the BBB. Results from the phase I OPARATIC trial (NCT01390571) in recurrent GBM patients showed that olaparib penetrated all tumor core and tumor margin specimens, even though preclinical studies had shown a lack of penetration of the intact BBB.^40^ The PARADIGM and PARADIGM-2 trials are currently investigating the safety and efficacy of olaparib and IR with or without TMZ in newly diagnosed GBM patients based on their MGMT status.^41^ In preclinical studies, the PARP inhibitor niraparib (45 mg/kg) exhibited better BBB penetration than olaparib and resulted in a 53% tumor growth inhibition compared to 27% with olaparib (75 mg/kg) after 44 days of treatment.^42^ Additionally, veliparib showed promising results in preclinical evaluation, however, results from the randomized phase II VERTU trial, did not provide any significant benefit (median survival 12.8 months vs 12.7 months) for unmethylated MGMT patients when given in combination with TMZ and radiotherapy.^43^ Compared to other PARP inhibitors, veliparib has demonstrated to have low potency and low PARP trapping activity. Although the strong PARP trapper talazoparib has shown to sensitize GBM cell lines and orthotropic GBM models to TMZ, it has demonstrated a restricted ability to cross the BBB.^44^ A phase II trial of talazoparib is currently recruiting recurrent high-grade glioma patients with DDR deficiency to investigate if combining carboplatin and radiation may sensitize tumors to PARP inhibition and increase talazoparib drug penetration across the BBB (NCT04740190).

### WEE1 Kinase Inhibitors

WEE1 is a nuclear enzyme that belongs to the Ser/Thr family of protein kinases. It functions as a G2/M checkpoint regulator and controls G2/M progression by phosphorylating and inactivating CDC25. Moreover, cells with mutated TP53 have a dysregulated G1/S cell cycle checkpoint and therefore rely on the G2/M checkpoint to induce cell arrest and repair damaged DNA.^45^ Consequently, WEE1 inhibition in p53-deficient tumor cells induces synthetic lethality.^46^ Indeed, in U251MG GBM cells it has been observed that genetic knockdown of WEE1 abolished IR and TMZ-induced cell cycle arrest in the G2/M phase, and eradicated brain tumors in mice when given in combination with IR.^47^ A similar effect was observed with the WEE1 inhibitor PD0166285 which was shown to radiosensitize U87MG, U118MG, U251MG, and U373MG cells, with enhancement ratios ranging from 1.19-1.95. Furthermore, PD0166285 enhanced the ability of IR to eradicate GBM neurospheres, and CD133 positive GBM cells are thought to represent GSCs.^47^ Of the WEE-1 inhibitors, AZD1775 (adavosertib) has shown increased radiosensitization across the broadest range of cancer models. Despite AZD1775 displaying poor BBB penetration in a GBM xenograft model, a phase 0 trial (NCT02207010) in recurrent GBM showed that unbound AZD1775 reached therapeutic concentrations within the tumor.^48^ This study also found that the patients treated with 200mg of AZD1775 demonstrated an ~8-fold increase in DNA DSBs, increased cell cycle abrogation, and an ~3-fold increase in apoptosis posttreatment. A phase I trial (NCT01849146) is currently investigating the safety and activity of AZD1775 in combination with TMZ and IR in newly diagnosed and recurrent GBM.

## Alternative Radiotherapy Treatments for GBM

### Proton Beam Therapy

Proton beam therapy (PBT), unlike conventional radiotherapy using photons (X-rays), utilizes positively charged proton ions to treat tumors and offers significant dosimetric benefits for some cancer types because of their physical characteristics. Specifically, proton beams exhibit low entrance radiation doses and as the protons lose energy and come to a stop, the maximum dose is released in a well-defined region known as the Bragg Peak, with no accompanying exit dose. In certain contexts, this allows larger doses of radiation to be administered directly to the tumor than is possible with photons, whilst sparing the associated normal tissues and organs at risk.

Evidence is accruing to support the concept that the neurocognitive complications of brain irradiation can be alleviated by PBT. Results from a prospective phase II trial indicated that GBM patients who received PBT had a significantly lower number of grade 2 or higher toxicities compared to patients who received intensity-modulated radiotherapy (IMRT).^49^ Whilst there is growing awareness that partial brain photon radiotherapy is associated with volume loss in both gray and white matter structures throughout the brain, early reports indicate that these effects are less pronounced after PBT.^50^ These observations are in keeping with results from a randomized phase III trial which showed a reduced neurocognitive decline in brain tumor patients treated with stereotactic compared with conventional photon radiotherapy.^51^ Around 40% of patients treated with the standard GBM treatment of radiation plus TMZ develop radiation-induced lymphopenia (RIL), which is adversely associated with disease-specific survival.^52^ RIL or decreased lymphocyte count is an independent predictor of poor clinical outcome and is associated with higher rates of relapse and infections, reduced response to treatments and decreased efficacy of immunotherapy.^53^ A phase II randomized trial investigated the effect of proton vs photon radiotherapy (with concomitant TMZ) in 28 and 56 GBM patients, respectively and reported reduced grade III lymphopenia with PBT.^54^

Although there is not yet any clear evidence that PBT improves tumor control and overall survival compared with conventional radiotherapy, in our view the dosimetric advantages of protons have not yet been exploited to the full. In particular, the use of advanced imaging modalities to identify regions at the highest risk of treatment failure in combination with particle radiation (IMPT) may enable meaningful dose escalation, with the potential to improve survival, while sparing normal tissues and reducing neurocognitive complications.

**Figure 2.**
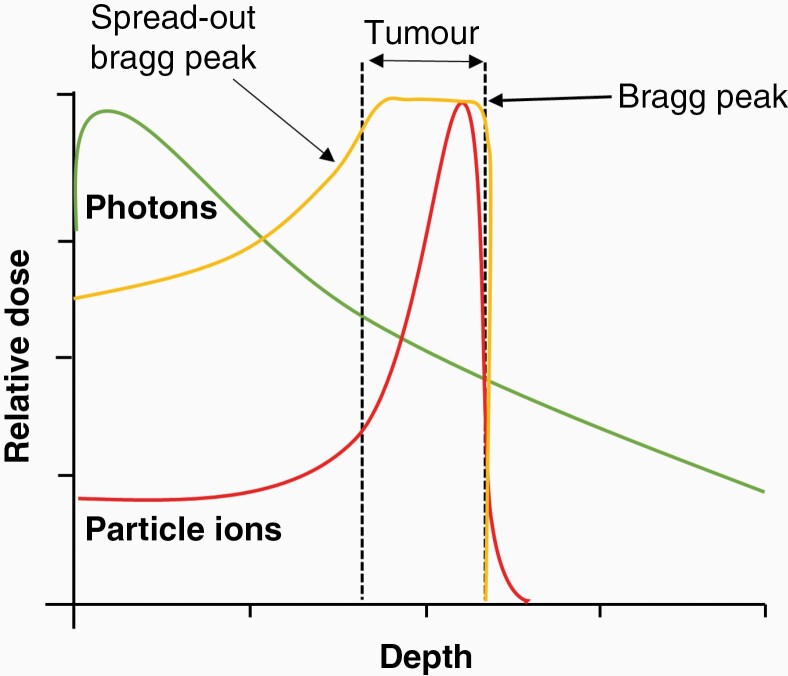
Dose distribution profile of photon vs particle ion radiotherapy. Photons (green lines) deposit the majority of energy at the entrance into the tissues. Particle ions (red line), enter with a low dose and deposit the maximum dose at the Bragg Peak positioned within the target tumor, which ultimately reduces exposure to normal tissues and organs at risk. Clinically, a spread-out Bragg peak (SOBP; yellow line) is used to widen the treatment area which is achieved by varying the energies of the incident beam.

In the clinical setting, a spread-out Bragg peak is used to irradiate larger tumor volumes, which is achieved by using a combination of beams with differential initial energies (Figure 2). Despite enhanced tumor targeting, PBT also displays significant biological uncertainty due to increases in linear energy transfer (LET) at and around the Bragg peak that is associated with changes in the DNA damage profile and particularly increases in the frequency and complexity of DNA damage.^8^ CDD is defined as 2 or more DNA lesions within one helical turn of the DNA and broadly can be considered as either non-DSB or DSB-containing CDD. This type of damage is considered the most difficult for the cells to repair and therefore contributes greatly to IR-induced cell death. Clinically, the relative biological effectiveness (RBE) of PBT compared to photons is assumed to be 1.1 (10% more effective). However, this is only an approximation and widely variable RBEs have been documented both *in vitro* and *in vivo* and depend on a number of biological (eg, tumor model and inherent radiosensitivity) and physical (eg, dose, dose rate, and LET) factors.^55,56^

Preclinical data pertaining to the impact of protons vs photons in GBM tumor models are very limited. Utilizing patient-derived GSCs, it has been reported that PBT (at mid-SOBP) led to significant reductions in cell survival compared to X-rays, and increased the number of SSB and DSBs several hours post-IR.^57^ This suggested that the DNA damage induced by PBT is different and has slow repair kinetics, although it is noticeable from the data that the GSCs were irradiated with a relatively high dose (10 Gy) that caused significant cell death, so this conclusion is questionable. It was also suggested that PBT exposure in GSCs led to increased reactive oxygen species levels, which enhanced cell death post-IR. These findings support the theory that RBE is enhanced with PBT vs photon radiotherapy in GSCs, although substantial further evidence is required to validate this. Nevertheless, there are several ongoing clinical trials assessing the efficacy of both PBT and CIRT in GBM patients (Table 1). The NCT04536649 trial is comparing the efficacy between 3 treatment groups (photon vs PBT vs CIRT boost and PBT), in newly diagnosed GBM patients to assess improvement in overall survival. The GRIPS randomized open-label phase III trial is assessing cumulative toxicity between PBT and photons and the effects of dose escalation are being investigated in the NCT02179086 trial.

**Table 1.** Ongoing Proton and Carbon Ion Radiotherapy Clinical Trials in GBM

Clinical status	NCT Number	Study	Initiated	Completion date/Estimated
Phase III	NCT04536649	Proton and carbon ion beam radiation vs photon beam radiation for newly diagnosed GBM	2020	2023
Phase II (CLEOPATRA)	NCT01165671	Carbon ion boost applied, after combined radiochemotherapy with TMZ vs a proton boost, after radiochemotherapy with TMZ in patients with primary GBM	2010	completed
Phase II	NCT01854554	IMPT vs IMRT in GBM	2013	completed
Phase III (GRIPS)	NCT04752280	IMRT vs proton radiotherapy in GBM	2021	2027
Phase II	NCT02179086	Hypofractionated dose-escalated photon IMRT or PBT vs standard photon irradiation with concomitant and adjuvant TMZ in patients with newly diagnosed GBM	2014	
Phase II	NCT03778294	18F-DOPA-PET/MRI scan in imaging elderly patients with newly diagnosed grade iv malignant glioma or GBM during planning for a short course of proton beam radiation therapy	2019	
Pilot study	NCT03587038	OKN-007 in combination with adjuvant TMZ chemoradiotherapy for newly diagnosed GBM	2018	2025
Open Label	NCT0528463	Spectroscopic MRI, proton therapy, and Avastin for glioblastoma patients	2022	

**Abbreviations:** GBM, glioblastoma; TMZ, temozolomide; IMPT, Intensity-modulated proton radiotherapy; IMRT, Intensity-modulated radiotherapy; MRI, Magnetic resonance imaging.

### Carbon Ion Radiotherapy

Although carbon ion radiotherapy (CIRT) shows a similar dose distribution profile to PBT, with the majority of the radiation dose being delivered to the target tumor via the Bragg Peak (Figure 2), it benefits from a significantly higher RBE (between 3–5) and which is not dependent on oxygen for radiosensitization. The densely ionizing track structure of CIRT causes more irreparable DNA damage that drives cell death. Despite this and similar to PBT, there is a distinct lack of preclinical evidence to examine the impact of CIRT in GBM cells. In a study in U87 cells *in vitro*, CIRT was found to be significantly more cytotoxic than photons (RBE = 3.3–3.9), and this effect was further exacerbated in combination with chemotherapeutic drugs, in particular paclitaxel and camptothecin.^58^ In another study CIRT has also been shown effective in the killing of X-ray resistant GSCs, with average RBE values ranging between 1.87 and 3.44.^59^ This increase in radiosensitivity of the GSCs was attributed to the failure of the cells to repair the DNA damage induced by CIRT, as reflected by the presence of residual unrepaired DNA DSBs 24 hours posttreatment. In terms of clinical observations, GBM patients have been treated with CIRT both as primary radiotherapy and as re-irradiations of recurrent tumors. CIRT was reportedly well tolerated with minimal toxicity reported.^60,61^ A phase I/II study in GBM patients compared the effects of X-rays to a dose equivalent to CIRT, and reported significant increases in survival from 4 to 7 months (low dose), 7 to 19 months (middle dose), and from 14 to 26 months (high dose) respectively.^62^ These non-randomized data must however be treated with caution. Currently, 13 centers around the world, with a large proportion located in Japan, use CIRT for GBM patients and several clinical trials are assessing its effectiveness (Table 1).

### Combining DDR Inhibitors With PBT and CIRT

As mentioned above, the number of preclinical studies analyzing responses of GBM tumor models to PBT and CIRT is very small. Therefore, strategies leading to optimal radiosensitization of GBM cells with these radiation modalities, such as through targeting the DDR, have not been investigated in any detail. In fact, while the level and complexity of CDD induced by PBT (relatively low-LET) and CIRT (high-LET) are known to be different, the relative importance of the various DDR pathways (eg, BER, NHEJ, and HR) that respond to this damage is still subject to debate. In tumor cell types other than GBM, it has been reported that most of the damage induced by CIRT was subjected to end resection, coordinated by CtIP and the MRN exonuclease.^63,64^ It was also suggested that there was preferential activation of the microhomology-mediated end joining (MHEJ) pathway over NHEJ in the repair of complex DSBs induced by CIRT in G1 phase cells. However, other studies have reported HR to play a prominent role in the repair of CDD induced by carbon ions, with NHEJ being employed in response to both PBT and CIRT.^65^ Another study reported that enhanced radiosensitivity to CIRT was more pronounced in cells with NHEJ deficiency, rather than in those with HR deficiency.^66^

In terms of PBT, it has been shown that U2OS and BT549 cells irradiated at the Bragg peak with a higher RBE upregulate utilization of HR, but interestingly that inhibiting ATM redirected cells to the NHEJ pathway leading to the formation of toxic DSB repair end products.^67^ This evidence was replicated in an *in vivo* xenograft model, in which inhibiting ATM in combination with PBT was shown to significantly delay tumor growth, and that Bragg peak protons were furthermore highly effective in the killing of HR-defective (BRCA-1 deficient) cells and tumors.^67^ Such findings suggest that cells may switch between different repair pathways when repairing more complex DSBs induced by high-LET radiations. Additionally, data acquired from our lab using HeLa and head and neck squamous cell carcinoma cells has demonstrated that relatively high-LET PBT generates CDD that triggers a specific cellular DDR response driven by histone H2B ubiquitylation, and that this damage is largely SSB-associated and requires PARP1 for repair.^68,69^ Indeed, using both PARP1 siRNA and the PARP inhibitor olaparib, we demonstrated specific radiosensitization of cells following relatively high-LET PBT, but not those irradiated with low-LET PBT at the entrance dose. In contrast, we showed that inhibitors of ATM, ATR, and DNA-PK enhance the sensitivity of head and neck cancer cells to both low-LET PBT and X-rays.^70^ Therefore, given the increasing use of PBT and CIRT in the clinic, it is possible that different DDR inhibitors should be used as radiosensitizers specifically for GBM in the context of different radiation modalities. It is also clear that substantially more preclinical studies are needed in clinically relevant GBM models if we are to identify the best strategies for prolonged tumor control and future patient benefit.

## Conclusions

Clinically, treatment of GBM is challenging due to its aggressive and therapy-resistant nature. Radiotherapy is the mainstay of GBM treatment, with chemotherapy options being limited partly by adverse pharmacokinetics. TMZ is the only drug clinically approved as a radiosensitizer but has minimal efficacy in the large subset of tumors in which the MGMT gene promoter is unmethylated. The radioresistant and hypoxic nature of GBM further complicates treatment, as does the molecular and phenotypic heterogeneity of the disease. Preclinical studies have demonstrated that targeting the DDR, particularly in radioresistant GSCs, can enhance the efficacy of conventional photon radiotherapy in tumor cell killing. However, the use of PBT and CIRT in the treatment of GBM are emerging as attractive therapeutic options, with superior dosimetry which may potentially reduce the adverse side effects associated with photon radiation, such as treatment-induced lymphopenia, and improve the overall quality of life. Recent mechanistic studies raise the exciting possibility of combining PBT and CIRT with DDR inhibitors to overcome therapy resistance in GBM, and indicate that different radiation modalities should be paired with specific agents to maximize clinical benefit. As with any novel therapeutic approach in this heterogeneous tumor type, it is highly likely that subsets of patients will derive particular benefits from these new combinations. Biomarkers that enable the selection of patients with responsive tumors are required to maximize the benefits of novel treatments, and these should be identified and validated as part of ongoing research in this field.

## References

[1] Patel AP , Fisher JL, Nichols E, et al. Global, regional, and national burden of brain and other CNS cancer, 1990-2016: a systematic analysis for the Global Burden of Disease Study 2016. Lancet Neurol. 2019;18(4):376–393. 30797715 10.1016/S1474-4422(18)30468-X PMC6416167

[2] Louis DN , Perry A, Wesseling P, et al The 2021 WHO classification of tumors of the central nervous system: a summary. Neuro Oncology. 2021;23(8):1231–1251. 34185076 10.1093/neuonc/noab106 PMC8328013

[3] Lee DH , Ryu HW, Won HR, Kwon SH. Advances in epigenetic glioblastoma therapy. Oncotarget. 2017;8(11):18577–18589. 28099914 10.18632/oncotarget.14612 PMC5392350

[4] Yabroff KR , Harlan L, Zeruto C, Abrams J, Mann B. Patterns of care and survival for patients with glioblastoma multiforme diagnosed during 2006. Neuro Oncol. 2012;14(3):351–359. 22241797 10.1093/neuonc/nor218 PMC3280803

[5] Lawrence YR , Mishra MV, Werner-Wasik M, et al Improving prognosis of glioblastoma in the 21st century: who has benefited most? Cancer. 2012;118(17):4228–4234. 22180310 10.1002/cncr.26685

[6] Weller M , Cloughesy T, Perry JR, Wick W. Standards of care for treatment of recurrent glioblastoma--are we there yet? Neuro Oncology. 2013;15(1):4–27. 23136223 10.1093/neuonc/nos273 PMC3534423

[7] Hegi ME , Diserens A-C, Gorlia T, et al MGMT gene silencing and benefit from temozolomide in glioblastoma. N Engl J Med. 2005;352(10):997–1003. 15758010 10.1056/NEJMoa043331

[8] Vitti ET , Parsons JL. The Radiobiological effects of proton beam therapy: impact on DNA damage and repair. Cancers. 2019;11(7):946. 10.3390/cancers11070946 PMC6679138 31284432

[9] Carruthers R , Ahmed SU, Strathdee K, et al Abrogation of radioresistance in glioblastoma stem-like cells by inhibition of ATM kinase. Mol Oncol. 2015;9(1):192–203. 25205037 10.1016/j.molonc.2014.08.003 PMC5528679

[10] Ahmed SU , Carruthers R, Gilmour L, et al Selective inhibition of parallel DNA damage response pathways optimizes radiosensitization of glioblastoma stem-like cells. Cancer Res. 2015;75(20):4416–4428. 26282173 10.1158/0008-5472.CAN-14-3790

[11] Balbous A , Cortes U, Guilloteau K, et al A radiosensitizing effect of RAD51 inhibition in glioblastoma stem-like cells. BMC Cancer. 2016;16:604. 27495836 10.1186/s12885-016-2647-9 PMC4974671

[12] MacLeod G , Bozek DA, Rajakulendran N, et al Genome-wide CRISPR-Cas9 screens expose genetic vulnerabilities and mechanisms of temozolomide sensitivity in glioblastoma stem cells. Cell Rep. 2019;27(3):971–986.e979. 30995489 10.1016/j.celrep.2019.03.047

[13] Tamura K , Aoyagi M, Ando N, et al Expansion of CD133-positive glioma cells in recurrent de novo glioblastomas after radiotherapy and chemotherapy. J Neurosurg. 2013;119(5):1145–1155. 23991844 10.3171/2013.7.JNS122417

[14] Hassn Mesrati M , Behrooz AB, Abuhamad A Y, Syahir A. Understanding glioblastoma biomarkers: knocking a mountain with a hammer. Cells. 2020;9(5):1236. 10.3390/cells9051236 PMC7291262 32429463

[15] Lee Y , Kim KH, Kim DG, et al FoxM1 promotes stemness and radio-resistance of glioblastoma by regulating the master stem cell regulator Sox2. PLoS One. 2015;10(10):e0137703–e0137703. 26444992 10.1371/journal.pone.0137703 PMC4596841

[16] Wang J , Wakeman TP, Lathia JD, et al Notch promotes radioresistance of glioma stem cells. Stem Cells. 2010;28(1):17–28. 19921751 10.1002/stem.261 PMC2825687

[17] Han N , Hu G, Shi L, et al Notch1 ablation radiosensitizes glioblastoma cells. Oncotarget. 2017;8(50):88059–88068. 29152141 10.18632/oncotarget.21409 PMC5675693

[18] Vaupel P , Höckel M, Mayer A. Detection and characterization of tumor hypoxia using pO2 histography. Antioxid Redox Signal. 2007;9(8):1221–1235. 17536958 10.1089/ars.2007.1628

[19] Chédeville AL , Madureira PA. The role of hypoxia in glioblastoma radiotherapy resistance. Cancers. 2021;13(3):542. 33535436 10.3390/cancers13030542 PMC7867045

[20] Monteiro AR , Hill R, Pilkington GJ, Madureira PA. The role of hypoxia in glioblastoma invasion. Cells. 2017;6(4):45. 10.3390/cells6040045 PMC5755503 29165393

[21] Albert I , Hefti M, Luginbuehl V. Physiological oxygen concentration alters glioma cell malignancy and responsiveness to photodynamic therapy in vitro. Neurol Res. 2014;36(11):1001–1010. 24923209 10.1179/1743132814Y.0000000401

[22] Brown JM , Giaccia AJ. The unique physiology of solid tumors: opportunities (and problems) for cancer therapy. Cancer Res. 1998;58(7):1408–1416. 9537241

[23] Park JS , Kim IK, Han S, et al Normalization of tumor vessels by Tie2 activation and Ang2 inhibition enhances drug delivery and produces a favorable tumor microenvironment. Cancer Cell. 2016;30(6):953–967. 27960088 10.1016/j.ccell.2016.10.018

[24] Moeller BJ , Cao Y, Li CY, Dewhirst MW. Radiation activates HIF-1 to regulate vascular radiosensitivity in tumors: role of reoxygenation, free radicals, and stress granules. Cancer Cell. 2004;5(5):429–441. 15144951 10.1016/s1535-6108(04)00115-1

[25] Kreisl TN , Kim L, Moore K, et al Phase II trial of single-agent bevacizumab followed by bevacizumab plus irinotecan at tumor progression in recurrent glioblastoma. J Clin Oncol. 2009;27(5):740–745. 19114704 10.1200/JCO.2008.16.3055 PMC2645088

[26] Simpson JE , Gammoh N. The impact of autophagy during the development and survival of glioblastoma. Open Biol. 2020;10(9):200184–200184. 32873152 10.1098/rsob.200184 PMC7536068

[27] Lomonaco SL , Finniss S, Xiang C, et al The induction of autophagy by gamma-radiation contributes to the radioresistance of glioma stem cells. Int J Cancer. 2009;125(3):717–722. 19431142 10.1002/ijc.24402

[28] Zheng W , Chen Q, Wang C, et al Inhibition of Cathepsin D (CTSD) enhances radiosensitivity of glioblastoma cells by attenuating autophagy. Mol Carcinog. 2020;59(6):651–660. 32253787 10.1002/mc.23194

[29] Zhou W , Yao Y, Scott AJ, et al Purine metabolism regulates DNA repair and therapy resistance in glioblastoma. Nat Commun. 2020;11(1):3811. 32732914 10.1038/s41467-020-17512-x PMC7393131

[30] Wang X , Yang K, Xie Q, et al Purine synthesis promotes maintenance of brain tumor initiating cells in glioma. Nat Neurosci. 2017;20(5):661–673. 28346452 10.1038/nn.4537 PMC6015494

[31] Taïb B , Aboussalah AM, Moniruzzaman M, et al Lipid accumulation and oxidation in glioblastoma multiforme. Sci Rep. 2019;9(1):19593. 31863022 10.1038/s41598-019-55985-z PMC6925201

[32] van den Bosch M , Bree RT, Lowndes NF. The MRN complex: coordinating and mediating the response to broken chromosomes. EMBO Rep. 2003;4(9):844–849. 12949583 10.1038/sj.embor.embor925 PMC1326362

[33] Vecchio D , Daga A, Carra E, et al Pharmacokinetics, pharmacodynamics and efficacy on pediatric tumors of the glioma radiosensitizer KU60019. Int J Cancer. 2015;136(6):1445–1457. 25091220 10.1002/ijc.29121

[34] Durant ST , Zheng L, Wang Y, et al The brain-penetrant clinical ATM inhibitor AZD1390 radiosensitizes and improves survival of preclinical brain tumor models. Sci Adv. 2018;4(6):eaat1719. 29938225 10.1126/sciadv.aat1719 PMC6010333

[35] Eich M , Roos WP, Nikolova T, Kaina B. Contribution of ATM and ATR to the resistance of glioblastoma and malignant melanoma cells to the methylating anticancer drug temozolomide. Mol Cancer Ther. 2013;12(11):2529–2540. 23960094 10.1158/1535-7163.MCT-13-0136

[36] Talele S , Zhang W, Burgenske DM, et al Brain distribution of berzosertib: an ataxia telangiectasia and Rad3-related protein inhibitor for the treatment of glioblastoma. J Pharmacol Exp Ther. 2021;379(3):343–357. 34556535 10.1124/jpet.121.000845 PMC9351722

[37] Lan T , Zhao Z, Qu Y, et al Targeting hyperactivated DNA-PKcs by KU0060648 inhibits glioma progression and enhances temozolomide therapy via suppression of AKT signaling. Oncotarget. 2016;7(34):55555–55571. 27487130 10.18632/oncotarget.10864 PMC5342436

[38] Zenke FT , Zimmermann A, Sirrenberg C, et al Pharmacologic inhibitor of DNA-PK, M3814, potentiates radiotherapy and regresses human tumors in mouse models. Mol Cancer Ther. 2020;19(5):1091–1101. 32220971 10.1158/1535-7163.MCT-19-0734

[39] Cowan MJ , Gennery AR. Radiation-sensitive severe combined immunodeficiency: the arguments for and against conditioning before hematopoietic cell transplantation--what to do? J Allergy Clin Immunol. 2015;136(5):1178–1185. 26055221 10.1016/j.jaci.2015.04.027 PMC4641002

[40] Hanna C , Kurian KM, Williams K, et al Pharmacokinetics, safety, and tolerability of olaparib and temozolomide for recurrent glioblastoma: results of the phase I OPARATIC trial. Neuro Oncology. 2020;22(12):1840–1850. 32347934 10.1093/neuonc/noaa104 PMC7746945

[41] Chalmers AJ , Short S, Watts C, et al Phase I clinical trials evaluating olaparib in combination with radiotherapy (RT) and/or temozolomide (TMZ) in glioblastoma patients: results of OPARATIC and PARADIGM phase I and early results of PARADIGM-2. J Clin Oncol. 2018;36(suppl 15):2018–2018.

[42] Sun K , Mikule K, Wang Z, et al A comparative pharmacokinetic study of PARP inhibitors demonstrates favorable properties for niraparib efficacy in preclinical tumor models. Oncotarget. 2018;9(98):37080–37096. 30647846 10.18632/oncotarget.26354 PMC6324689

[43] Sim HW , McDonald KL, Lwin Z, et al A randomized phase II trial of veliparib, radiotherapy, and temozolomide in patients with unmethylated MGMT glioblastoma: the VERTU study. Neuro Oncol. 2021;23(10):1736–1749. 33984151 10.1093/neuonc/noab111 PMC8485443

[44] Kizilbash SH , Gupta SK, Chang K, et al Restricted delivery of talazoparib across the blood-brain barrier limits the sensitizing effects of PARP inhibition on temozolomide therapy in glioblastoma. Mol Cancer Ther. 2017;16(12):2735–2746. 28947502 10.1158/1535-7163.MCT-17-0365 PMC5716902

[45] Petitjean A , Mathe E, Kato S, et al Impact of mutant p53 functional properties on TP53 mutation patterns and tumor phenotype: lessons from recent developments in the IARC TP53 database. Hum Mutat. 2007;28(6):622–629. 17311302 10.1002/humu.20495

[46] Leijen S , Beijnen JH, Schellens JH. Abrogation of the G2 checkpoint by inhibition of Wee-1 kinase results in sensitization of p53-deficient tumor cells to DNA-damaging agents. Curr Clin Pharmacol. 2010;5(3):186–191. 20406171 10.2174/157488410791498824

[47] Mir SE , De Witt Hamer PC, Krawczyk PM, et al In silico analysis of kinase expression identifies WEE1 as a gatekeeper against mitotic catastrophe in glioblastoma. Cancer Cell. 2010;18(3):244–257. 20832752 10.1016/j.ccr.2010.08.011 PMC3115571

[48] Sanai N , Li J, Boerner J, et al Phase 0 trial of AZD1775 in first-recurrence glioblastoma patients. Clin Cancer Res. 2018;24(16):3820–3828. 29798906 10.1158/1078-0432.CCR-17-3348 PMC6865048

[49] Brown PD , Chung C, Liu DD, et al A prospective phase II randomized trial of proton radiotherapy vs intensity-modulated radiotherapy for patients with newly diagnosed glioblastoma. Neuro Oncol. 2021;23(8):1337–1347. 33647972 10.1093/neuonc/noab040 PMC8328012

[50] Petr J , Platzek I, Hofheinz F, et al Photon vs. proton radiochemotherapy: effects on brain tissue volume and perfusion. Radiother Oncol. 2018;128(1):121–127. 29370984 10.1016/j.radonc.2017.11.033

[51] Jalali R , Gupta T, Goda JS, et al Efficacy of stereotactic conformal radiotherapy vs conventional radiotherapy on benign and low-grade brain tumors: a randomized clinical trial. JAMA Oncol. 2017;3(10):1368–1376. 28570730 10.1001/jamaoncol.2017.0997 PMC5710529

[52] Grossman SA , Ye X, Lesser G, et al Immunosuppression in patients with high-grade gliomas treated with radiation and temozolomide. Clin Cancer Res. 2011;17(16):5473–5480. 21737504 10.1158/1078-0432.CCR-11-0774 PMC3156964

[53] Yovino S , Grossman SA. Severity, etiology and possible consequences of treatment-related lymphopenia in patients with newly diagnosed high-grade gliomas. CNS Oncol. 2012;1(2):149–154. 23828734 10.2217/cns.12.14 PMC3697135

[54] Mohan R , Liu AY, Brown PD, et al Proton therapy reduces the likelihood of high-grade radiation-induced lymphopenia in glioblastoma patients: phase II randomized study of protons vs photons. Neuro Oncol. 2020;23(2):284–294. 10.1093/neuonc/noaa182 PMC7906048 32750703

[55] Paganetti H . Relative biological effectiveness (RBE) values for proton beam therapy. Variations as a function of biological endpoint, dose, and linear energy transfer. Phys Med Biol. 2014;59(22):R419–R472. 25361443 10.1088/0031-9155/59/22/R419

[56] Paganetti H , van Luijk P. Biological considerations when comparing proton therapy with photon therapy. Semin Radiat Oncol. 2013;23(2):77–87. 23473684 10.1016/j.semradonc.2012.11.002

[57] Mitteer RA , Wang Y, Shah J, et al Proton beam radiation induces DNA damage and cell apoptosis in glioma stem cells through reactive oxygen species. Sci Rep. 2015;5:13961. 26354413 10.1038/srep13961 PMC4564801

[58] Combs SE , Zipp L, Rieken S, et al In vitro evaluation of photon and carbon ion radiotherapy in combination with chemotherapy in glioblastoma cells. Radiat Oncol. 2012;7(1):9. 22284807 10.1186/1748-717X-7-9 PMC3398277

[59] Chiblak S , Tang Z, Campos B, et al Radiosensitivity of patient-derived glioma stem cell 3-dimensional cultures to photon, proton, and carbon irradiation. Int J Radiat Oncol Biol Phys. 2016;95(1):112–119. 26254681 10.1016/j.ijrobp.2015.06.015

[60] Combs SE , Ellerbrock M, Haberer T, et al Heidelberg Ion Therapy Center (HIT): initial clinical experience in the first 80 patients. Acta Oncol. 2010;49(7):1132–1140. 20831505 10.3109/0284186X.2010.498432

[61] Rieken S , Habermehl D, Nikoghosyan A, et al Assessment of early toxicity and response in patients treated with proton and carbon ion therapy at the Heidelberg ion therapy center using the raster scanning technique. Int J Radiat Oncol Biol Phys. 2011;81(5):e793–e801. 21300464 10.1016/j.ijrobp.2010.12.018

[62] Mizoe JE , Tsujii H, Hasegawa A, et al Phase I/II clinical trial of carbon ion radiotherapy for malignant gliomas: combined X-ray radiotherapy, chemotherapy, and carbon ion radiotherapy. Int J Radiat Oncol Biol Phys. 2007;69(2):390–396. 17459607 10.1016/j.ijrobp.2007.03.003

[63] Yajima H , Fujisawa H, Nakajima NI, et al The complexity of DNA double strand breaks is a critical factor enhancing end-resection. DNA Repair. 2013;12(11):936–946. 24041488 10.1016/j.dnarep.2013.08.009

[64] Averbeck NB , Ringel O, Herrlitz M, et al DNA end resection is needed for the repair of complex lesions in G1-phase human cells. Cell Cycle. 2014;13(16):2509–2516. 25486192 10.4161/15384101.2015.941743 PMC4615131

[65] Gerelchuluun A , Manabe E, Ishikawa T, et al The major DNA repair pathway after both proton and carbon-ion radiation is NHEJ, but the HR pathway is more relevant in carbon ions. Radiat Res. 2015;183(3):345–356. 25738894 10.1667/RR13904.1 PMC5684887

[66] Takahashi A , Kubo M, Ma H, et al Nonhomologous end-joining repair plays a more important role than homologous recombination repair in defining radiosensitivity after exposure to High-LET radiation. Radiat Res. 2014;182(3):338–344. 25117625 10.1667/RR13782.1

[67] Zhou Q , Howard ME, Tu X, et al Inhibition of ATM induces hypersensitivity to proton irradiation by upregulating toxic end joining. Cancer Res. 2021;81(12):3333–3346. 33597272 10.1158/0008-5472.CAN-20-2960 PMC8260463

[68] Carter RJ , Nickson CM, Thompson JM, et al Complex DNA damage induced by high linear energy transfer alpha-particles and protons triggers a specific cellular DNA damage response. Int J Radiat Oncol Biol Phys. 2018;100(3):776–784. 29413288 10.1016/j.ijrobp.2017.11.012 PMC5796827

[69] Carter RJ , Nickson CM, Thompson JM, et al Characterisation of deubiquitylating enzymes in the cellular response to high-LET ionizing radiation and complex DNA damage. Int J Radiat Oncol Biol Phys. 2019;104(3):656–665. 30851349 10.1016/j.ijrobp.2019.02.053 PMC6542414

[70] Vitti ET , Kacperek A, Parsons JL. Targeting DNA double-strand break repair enhances radiosensitivity of HPV-positive and HPV-negative head and neck squamous cell carcinoma to photons and protons. Cancers. 2020;12(6):1490. 10.3390/cancers12061490 PMC7352833 32517381

